# Transfer RNA Bound to MnmH Protein Is Enriched with Geranylated tRNA – A Possible Intermediate in Its Selenation?

**DOI:** 10.1371/journal.pone.0153488

**Published:** 2016-04-13

**Authors:** Gunilla Jäger, Peng Chen, Glenn R. Björk

**Affiliations:** 1 Department of Molecular Biology, Umeå university, 901 87, Umeå, Sweden; 2 Biomass and Bioenergy Research Centre, College of Plant Sciences and Technology, Huazhong Agricultural University, Wuhan, 430070, P. R. China; Centro de Biología Molecular Severo Ochoa (CSIC-UAM), SPAIN

## Abstract

The wobble nucleoside 5-methylaminomethyl-2-thio-uridine (mnm^5^s^2^U) is present in bacterial tRNAs specific for Lys and Glu and 5-carboxymethylaminomethyl-2-thio-uridine (cmnm^5^s^2^U) in tRNA specific for Gln. The sulfur of (c)mnm^5^s^2^U may be exchanged by selenium (Se)–a reaction catalyzed by the selenophosphate-dependent tRNA 2-selenouridine synthase encoded by the *mnmH* (y*bbB*, *selU*, *sufY*) gene. The MnmH protein has a rhodanese domain containing one catalytic Cys (C97) and a P-loop domain containing a Walker A motif, which is a potential nucleotide binding site. We have earlier isolated a mutant of *Salmonella enterica*, serovar Typhimurium with an alteration in the rhodanese domain of the MnmH protein (G67E) mediating the formation of modified nucleosides having a geranyl (ge)-group (C_10_H_17_-fragment) attached to the s^2^ group of mnm^5^s^2^U and of cmnm^5^s^2^U in tRNA. To further characterize the structural requirements to increase the geranylation activity, we here report the analysis of 39 independently isolated mutants catalyzing the formation of mnm^5^ges^2^U. All these mutants have amino acid substitutions in the rhodanese domain demonstrating that this domain is pivotal to increase the geranylation activity. The wild type form of MnmH^+^ also possesses geranyltransferase activity *in vitro* although only a small amount of the geranyl derivatives of (c)mnm^5^s^2^U is detected *in vivo*. The selenation activity *in vivo* has an absolute requirement for the catalytic Cys97 in the rhodanese domain whereas the geranylation activity does not. Clearly, MnmH has two distinct enzymatic activities for which the rhodanese domain is pivotal. An intact Walker motif in the P-loop domain is required for the geranylation activity implying that it is the binding site for geranylpyrophosphate (GePP), which is the donor molecule *in vitro* in the geranyltransfer reaction. Purified MnmH from wild type and from the MnmH(G67E) mutant have bound tRNA, which is enriched with geranylated tRNA. This in conjunction with earlier published data, suggests that this bound geranylated tRNA may be an intermediate in the selenation of the tRNA.

## Introduction

Transfer RNA plays a pivotal role in translating the information residing in mRNA into proteins. The different species of tRNA are about 75–90 nucleotides in length and of all nucleic acids present in the cell, tRNA contains the highest proportion of modified nucleosides. These are derivatives of the four normal nucleosides adenosine (A), guanosine (G), uridine (U) and cytidine (C). At present more than 100 different modified nucleosides in tRNA from different organisms are characterized (http://mods.rna.albany.edu/ or http://modomics.genesilico.pl/). The synthesis of the modified nucleosides is catalyzed by highly specific enzymes using precursor tRNA molecules as substrates. Position 34 (the wobble nucleoside) and position 37 (the nucleoside adjacent and 3´of the anticodon) in tRNAs from all organisms are not only frequently modified (61% and 75%, respectively) but a large variety of different modified nucleosides are also present in these two positions [1]C:\GetARef\Refs\CTS_2012.ref #1;. These modified nucleosides in tRNA are important for a proper and efficient reading of the mRNA and lack of some of them results in severe physiological defects and/or no viability (Reviewed in [1]). Although several of these modified nucleosides have different chemical structures, are present in different tRNAs, and at different positions in the tRNA, many of them are important for keeping the ribosome in the correct reading frame [2].

The modified nucleosides (c)mnm^5^s^2^U34 in bacteria and mcm^5^s^2^U34 in eukaryotes are present in the wobble position of Gln-, Lys- and Glu-tRNA and presence of these modifications in tRNA is essential for viability in yeast [3] and in bacteria (unpublished observation). Deficiency of (c)mnm^5^s^2^U as well as hypermodification of tRNA (see below) in bacteria induces error in the reading frame maintenance, which is phenotypically manifested by ability to suppress +1 frameshift mutations. Lack of either the side chain in position 5 or the sulfur at position 2 induces frameshifts as well as addition of a geranyl group (C_10_H_17_-fragment) to the sulfur at position 2 of (c)mnm^5^s^2^U does [2, 4]. This geranylation reaction is catalyzed by the MnmH protein and the geranylation at the s^2^-group is increased by a mutation in the *mnmH* gene (earlier denoted *sufY*, *ybbB* or *selU*) resulting in an amino acid substitution G67E of MnmH [4]. Such +1 frameshift suppressors were first isolated by Riddle and Roth [5, 6] and later by Jäger *et al* [7]. The first isolated *mnmH* mutations are genetically dominant mediating the addition of a geranylgroup to the sulfur of cmnm^5^s^2^U of ${\text{tRNA}}_{\text{cmnm}5\text{s}2\text{UUG}}^{\text{Gln}}$ [4]. This was later confirmed by Dumelin *et al* [8] and these authors further showed that a geranyl group is also added to mnm^5^s^2^U in ${\text{tRNA}}_{\text{mnm}5\text{s}2\text{UUU}}^{\text{Lys}}$ and in ${\text{tRNA}}_{\text{mnm}5\text{s}2\text{UUC}}^{\text{Glu}}$ [8]. The G67E alteration of the MnmH increases the intrinsic low geranylation activity of the wild type MnmH [4, 8]. Earlier the MnmH (SelU, SufY, YbbB) protein was also shown to replace the sulfur of (c)mnm^5^s^2^U by selenium [9]. Clearly, the MnmH protein has two distinct enzymatic activities–a geranylation and a selenation activity.

**Fig 1:**
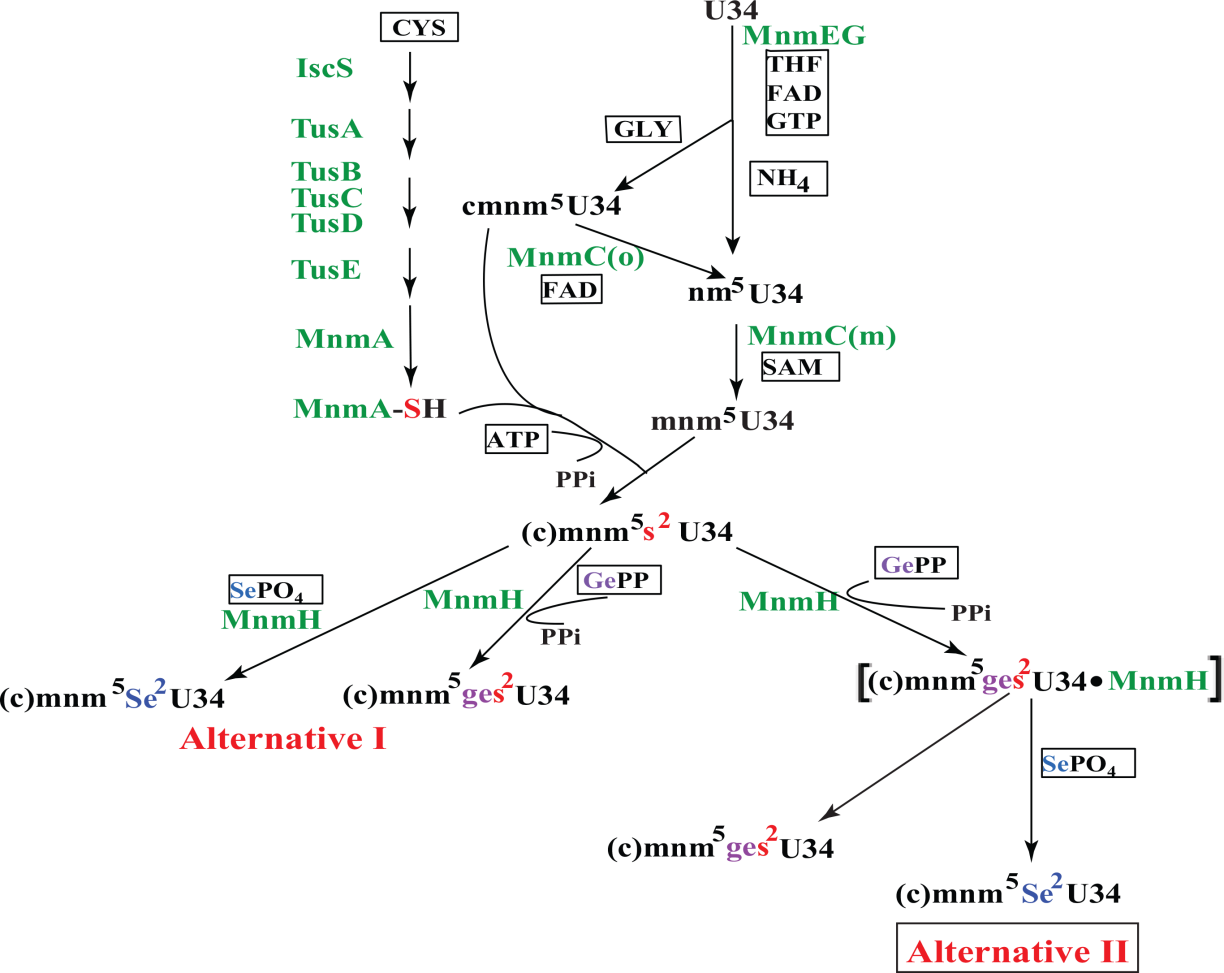
Synthesis of (c)mnm^5^s^2^U, (c)mnm^5^Se^2^U, and (c)mnm^5^ges^2^U (ge- is the abbreviation of a geranyl group). The sulfuration starts with IscS capturing sulfur from cysteine and through a sulfur relay system the sulfur is finally forming mnm^5^s^2^U or any other intermediate in the synthesis of the side chain in position 5 of U [11]. The synthesis of the latter starts with adding a C1 fragment catalyzed by the MnmEG complex. Thereafter two pathways occur depending on if ammonia or Gly is used as substrate by the MnmEG complex. The MnmC possesses two enzymatic activities–an oxidase activity (mnmC(o)) and a methyltransferase activity (MnmC(m)). MnmH may either directly exchange s^2^ of (c)mnm^5^s^2^U for Se or catalyze the geranylation of it using geranylpyrophosphate (GePP) as the donor of the geranyl group (Alt I). In Alternative II the geranylated derivative is an intermediate in the synthesis of the selenated derivative (c)mnm^5^Se^2^U. Note, in either alternatives the MnmH has two distinct enzymatic activities.

The synthesis of cmnm^5^s^2^U is complex and requires eleven different proteins of which two (MnmC and MnmH) have two enzymatic activities (Summarized in Fig 1; Reviewed recently in [1, 10]). The sulfur pathway depends on the IscS protein, which mobilizes the sulfur of cysteine which is then relayed to the wobble uridine in tRNA to form the (c)mnm^5^s^2^U [11]. The two pathways–synthesis of the (c)mnm^5^-side chain and the thiolation at position 2 –occur independently of each other; i.e lack of one modification does not influence the synthesis of the other. At a certain level and depending on the availability of selenium and geranylpyrophosphate (GePP) a fraction of the (c)mnm^5^s^2^U is selenated or geranylated. Two alternative metabolic pathways may be envisioned–the MnmH either selenates or geranylates the s^2^-position of cmnm^5^s^2^U (Alternative I) or the geranylated (c)mnm^5^s^2^U (abbreviated (c)mnm^5^ges^2^U; “ge-”is the abbreviation used for the geranyl group) is an intermediate in the selenation reaction creating the final product (c)mnm^5^Se^2^U (Alternative II). In Alternative I the MnmH protein might be a moonlighting protein provided that both these distinct activities resides in the same domain (one criteria for a moonlighting protein [12, 13]). In Alternative II the MnmH protein has two distinct activities operating in a linear biosynthetic pathway with the end product (c)mnm^5^Se^2^U. The MnmC protein also has two activities (an oxidase and a methyltransferase activity) operating in a linear biosynthetic pathway but these activities reside in two distinct domains.

**Fig 2:**
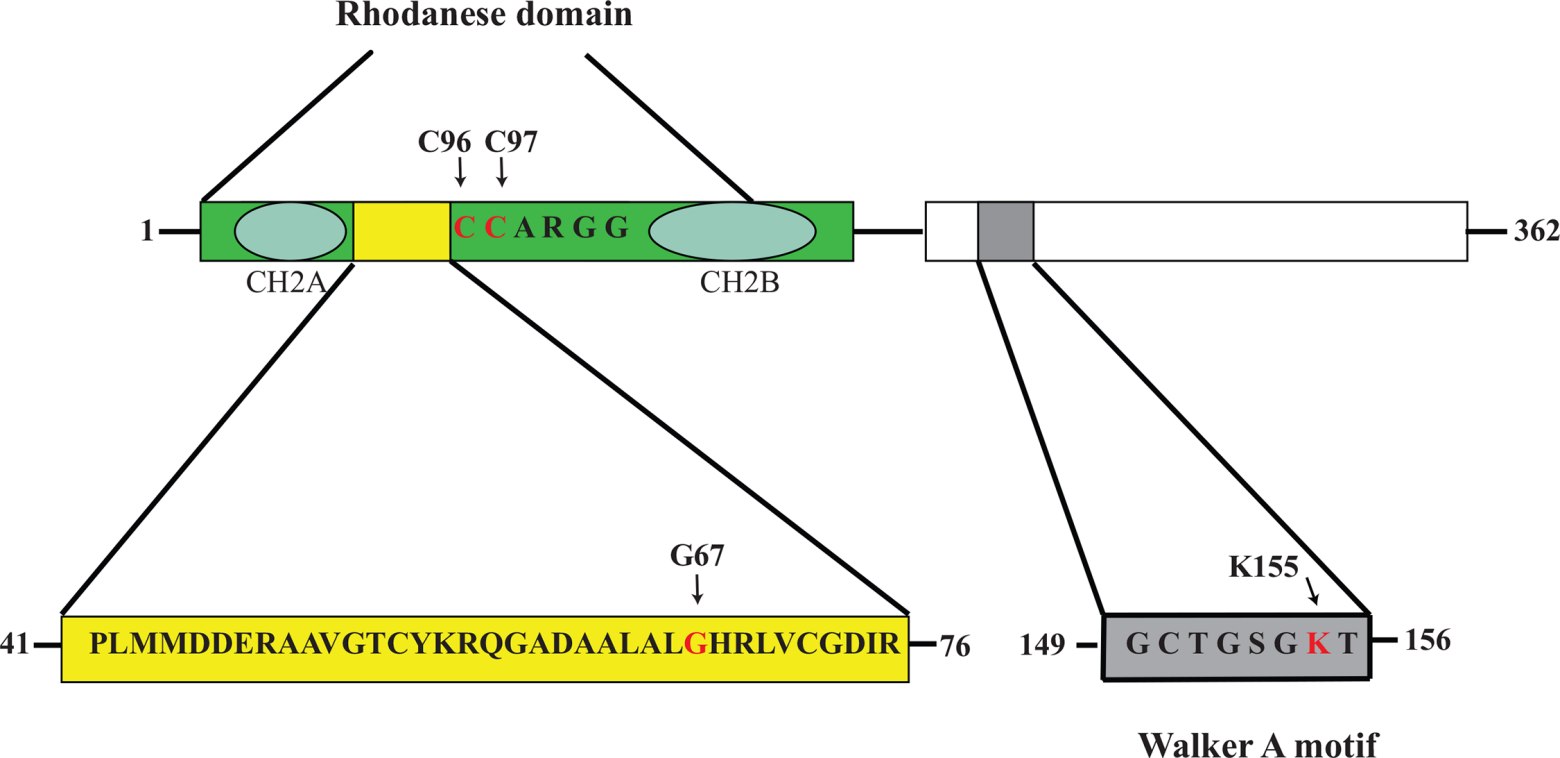
Domain organization of the MnmH protein from *S*. *enterica* (Modified from [9]). MnmH consists of two domains–a rhodanese domain with its Cys97 in the active center (Red indicates amino acids which has been altered by a site specific alterations) and a Walker A motif **G**CTGS**GKT** (a Walker A motif is G-x(4)-GKT [18, 19]), which is a binding site for ATP and GTP. A conserved 36 amino acid insertion present in the rhodanese domain of MnmH orthologues is not present in single-domain rhodaneses like GlpE [9]. The rhodanese signature motifs (CH2A and CH2B), the conserved active center (C97-XX-G) in rhodaneses, and the P-loop are shown. Position G67 is also shown since the first mutant form characterized of MnmH has an amino acid substitution (G67E) at this position.

The MnmH protein is composed of two domains–a rhodanese domain and a P-loop domain (Fig 2). Rhodaneses are ubiquitous proteins that catalyze the transfer of a sulfur atom from thiosulfate (rhodanese) or 3-mercaptopyruvate to e.g. cyanide *in vitro* (Reviewed in [14, 15]). The rhodaneses contain a conserved Cys (C97 in MnmH) that is absolutely required for their catalysis. In the single-domain rhodaneses this domain comprises the entire protein (e.g. GlpE) but such a domain can also be fused to other protein domains; e.g. the ThiI protein contains a rhodanese, a P-loop, and a THUMP domain and it catalyses both the biosynthesis of the thiazole ring of thiamine and the modified nucleoside 4-thiouridine (s^4^U) [16]. Whereas the two domains of bacterial MnmH are encoded by a single gene, the archaeal MnmH protein is encoded by two separated genes of which the gene encoding the P-loop is immediately upstream of the gene encoding the rhodanese domain [17]. Such a gene organization will result in the synthesis of two separate proteins and both of them are required to obtain selenation activity [17]. In both bacterial and archaeal orthologues of MnmH there is a conserved amino acid insertion of about 33 amino acids (36 amino acids in *Salmonella enterica* serovar Typhimurium, yellow in Fig 2), which is not present in a single domain rhodanese [9, 17]. The P-loop domain has a Walker A motif, which is present in many ATP or GTP binding proteins [18, 19]. This domain may also be responsible for the tRNA binding [9, 17]. A purified bacterial MnmH protein catalyses *in vitro* the replacement of sulfur by selenium without any other additions than tRNA, buffer, and SePO_4_ (the latter was made from HSe^-^ by selenium phosphate synthetase and ATP, which was included in the coupled reaction) and this replacement has a strict requirement *in vivo* for an intact catalytic C97 present in the rhodanese domain [9].

This paper focuses on the requirements for the geranylation reaction. We demonstrate that the increased geranylation activity resides in the rhodanese domain which is also pivotal to the selenation activities [9]. One possibility would be that selenation and geranylation occur in parallel catalysed by MnmH (Alternative I, Fig 1). We further show that the purified protein from various mutant forms of MnmH induces a strong binding of the chaperon GroEL whereas the wild type version of MnmH binds GroEL much less. Both the wild type form and all mutant forms of MnmH have tRNA strongly bound to them. Interestingly, the enzyme bound tRNA is enriched with the geranylated form of tRNA suggesting a possible role of geranylated derivative of (c)mnm^5^s^2^U as being an intermediate in the replacement of the sulfur by selenium (Alternative II, Fig 1).

## Materials and Method

### Bacteria and growth conditions

The bacterial strains used were derivatives of *Salmonella enterica* serovar Typhimurium or *Escherichia coli* K12 (S1 Table). As rich medium Luria-Bertani (LB) was used [20]C:\GetARef\Refs\Refsmanus\FS_mutant_2011.ref #2; C:\GetARef\Refs\Refsmanus\Kristina-Gln05.ref #38;.The minimal solid medium was made from the basal medium C:\GetARef\Refs\Refsmanus\FS_mutant_2011.ref #4; Vogel & Bonner [21]C:\GetARef\Refs\Refsmanus\Kristina-Gln05.ref #39; with 15g of agar per liter and supplemented with 0.2% glucose and required amino acids and/or vitamins [22]C:\GetARef\Refs\Refsmanus\FS_mutant_2011.ref #5; C:\GetARef\Refs\Refsmanus\Kristina-Gln05.ref #40;. When necessary antibiotics were added at following concentrations: carbenicillin 100 μg ml^-1^, kanamycin 50 μg ml^-1^ and chloramphenicol 25 μg ml^-1^

### Genetic procedures

Transduction with phage P22 HT105/1 (*int-201*) [23] C:\GetARef\Refs\Refsmanus\FS_mutant_2011.ref #20; was performed as previously described [22]C:\GetARef\Refs\Refsmanus\Kristina-Gln05.ref #40;. DNA sequencing was performed on plasmid DNA or PCR products following the manual of Applied Biosystems ABI PRISM Cycle Sequencing Ready Reaction Kit Big Dye^TM^ v.1.1 or by LightRun^TM^ sequencing at GATC Biotech, Cologna, Germany.

### Random mutagenization of the codon for G67 in MnmH

Plasmid pUST210 (pCL1921 harbouring the *mnmH*^+^ gene and a spectinomycin resistant marker) was amplified with two primers where nucleotides 199–201 (encoding G67) were scrambled creating codons for different amino acids. The resulting plasmids were transformed into strain JM109 supercompetent cells (Promega) and plated on agar plates containing LB and spectinomycin (100 μg /ml). 300 transformants were collected and used for preparing a pool of plasmids which was thereafter introduced into strain GT6874 (*ΔmnmH*, *hisO1242*, *hisC3737* (a +1 frameshift mutation in the *hisC* gene)). The transformation mixture was plated on medium E agar plates containing 0.2% glucose and spectinomycin (400 μg /ml) to select His^+^ Sp^R^ transformants. Thirty six such colonies were purified by restreaking and the *mnmH* gene on the plasmid was sequenced. To move the mutations to the chromosome a fragment with a tetracycline resistance marker was placed in the chromosomal located *mnmH* gene [24]. A fragment containing a mutation was amplified from the corresponding plasmid and this fragment was used in a linear transformation to replace the tetracycline resistant (Tc^R^) marker by selecting tetracycline sensitive (Tc^S^) clones [25, 26]**.** The mutated codon presence on the chromosome was confirmed by sequencing.

### Mutagenesis to obtain independently isolated mutants with an altered MnmH protein

Mutagenesis of the strain GT5688 (insertion *zbb-2523*::Tn*10d*Tc close to *mnmH*) was performed by inducing the expression of DinB [27, 28] from plasmid pSMP24 (Cb-R) [29],C:\GetARef\Refs\Refsmanus\Kristina-Gln05.ref #44; which harbor the *dinB* expressed from an *ara*-promoterC:\GetARef\Refs\Refsmanus\Kristina-Gln05.ref #43; C:\GetARef\Refs\Refsmanus\Kristina-Gln05.ref #42;. An over-night culture of strain GT5688 was diluted 2x10^6^-fold and inoculated into 500 μl of LB+100 μg carbenicillin (Cb)/ml +0.08% L-arabinose. After 24 hours of growth at 37°C phage P22 was added to make a phage stock. The phage lysates were used to infect strain GT7321 (*mnmH*^*+*^, *hisD10122*) containing a +1 frameshift mutation in the *hisD* gene. Following selection for tetracycline resistant (Tc^R^) colonies, His^+^ clones were selected among them. After a backcross the *mnmH* gene was sequenced to identify the mutation causing the His^+^ phenotype.

To mutagenize with hydroxylamine a phage P22 lysate prepared on strain GT5688 (insertion *zbb-2523*::Tn*10d*Tc close to *mnmH*) was treated with hydroxylamine as described [30]C:\GetARef\Refs\Jocke\proM.ref #26; until approximately 0.1 per cent infectious phage P22 particles remained. This lysate was used to transduce strain GT7321 (*hisD10122*; a +1 frameshift mutation in the *hisD* gene) and following selection for Tc^R^ clones, His+ clones were selected.

Nitrosoguanidine (NG) induced mutations were obtained by placing a crystal of NG on a lawn of the donor strain GT5688 (insertion *zbb-2523*::Tn*10d*Tc close to *mnmH*). Around the NG crystal a ring of growing bacteria emerged that contains bacteria with mutations induced by NG. Bacteria were scraped off from several fractions of the bacterial ring and suspended in 1 ml of LB and suspended cells were allowed to grow for 1–2 hours before phage P22 was added to make a lysate. As above the phage lysate was used to infect His^-^ strain harboring the +1 frameshift mutation *hisD10122*. Tc^R^ clones were selected and among them His^+^ clones were selected. To avoid siblings only one mutant from each phage stock was saved.

### Amino acid alterations of Cys96, Cys97, or K155 of MnmH

To create amino acid alterations at defined positions, a fragment bearing the mutation was transformed into a strain with a Tn*10d*Tc tetracycline resistance cassette in the *mnmH* gene. Tetracycline sensitive recombinants were selected on Bochner plates [25], purified, and tested for loss of Tc^R^. The presence of the desired mutation was confirmed by sequencing of Tc^S^ clones. The alteration of K155 to A was done by placing a Km^R^ cassette after the *mnmH* gene, and then amplifying a fragment with a primer harbouring the mutation as one primer and a primer binding in the cassette as the other primer. This fragment containing the mutation and Km^R^ was then transformed into LT2 (wt) and GT1484 (*mnmH204*(G67E)), recombinants were selected on kanamycin plates and Km^R^ clones were sequenced.

### Analysis of modified nucleosides in tRNA

Bacterial strains were grown over night in medium LB, diluted 100 times in 100 ml of the same medium and grown at 37°C to a cell density of about 4x10^8^ cells/ml. Cells were lysed and total RNA was prepared [31] and dissolved in 2 ml buffer R200 (10 mM Tris-H_3_PO_4_ (pH 6.3), 15% ethanol, 200 mM KCl) and applied to a Nucleobond^®^ AX500 column (Macherey-Nagel Gmbh & Co., Düren, Germany), pre-equilibrated with the same buffer. The column was washed once with 6 ml R200 and once with 2.0 ml R650 (same composition as R200, except for 650 mM KCl instead of 200 mM KCl). Finally, tRNA was eluted with 7 ml R650, precipitated by 0.7 volumes cold isopropanol, washed twice with 70% ethanol and dissolved in water. Transfer RNA was digested to nucleosides by nuclease P1 followed by treatment with bacterial alkaline phosphatase at pH 8.3 [32]. C:\GetARef\Refs\Jocke\cmo5U.ref #91; The hydrolysate was analyzed as described earlier [33] using a Supelcosil C-18 column (Supelco) or Develosil C-30 (Phenomenex) with a Waters Alliance HPLC system. The degradation method used results in nucleosides except if a complex nucleoside is present which usually gives a dinucleotide as a result. The complex geranylated derivatives are therefore present in the hydrolysate as a dinucleotide (NpN). Since geranylation of tRNA is occuring only on (c)mnm^5^s^2^U containg tRNAs, i.e. Gln, Lys and Glu specific tRNAs, there will always be a U35 next to the geranylated (c)mnm^5^s^2^U34. Therefore, the degradation by enzyme P1 results in a dinucleotide of the type ges^2^UpU. The separation of the various geranylated derivatives are shown in S1 Fig. The various ges^2^-derivatives were identified by their characteristic spectra and by MS analysis of their molecular weights ([4] and data not shown).

### Preparations of membranes

Membranes were prepared essentially as described by Wai et al [34] from 50 ml culture grown over night in LB Broth. The cells were washed with 10 mM Tris-HCl, pH 8.0, suspended in 1 ml of the same buffer and sonicated. The cell debris was pelleted by centrifugation at 5000 x g for 10 min and the resulting supernatant was centrifuged 30 min at 10000 x g. The sediment was used as the total membrane fraction. This fraction was separated into inner and outer membranes by adding 100 μl of 2% N-lauryl sarcosyl in 10 mM Tris buffer pH 8.0 and the mixture was incubated at room temperature for 30 min. After centrifugation for 30 min at 15000 x g the supernatant, which is the inner membrane fraction, was dialysed against 1000 ml 10 mM Tris-HCl, pH 8.0. The outer membrane in the pellet was suspended in 350 μl 10 mM Tris-HCl, pH 8.0 buffer and dialysed against the same buffer. Both outer and inner membrane fractions were extracted twice with phenol-chloroform-isoamylalcohol solution (25:24:1). The water phase, which contains RNA, was concentrated and loaded on a 12.5% urea PAGE gel in 0.5 M Tris-Borat-EDTA buffer. The gel was run for 1h at 200V and was blotted to Zetaprobe (Biorad) in a wet blot apparatus using the same buffer. As positive control total tRNA from strain GT8277 (*ΔmnmH*) was used. The tRNA was detected by Northern hybridization essentially as described earlier [35] using oligonucleotides labelled with [γ^32^P]–triphosphate (Perkin-Elmer). The oligonucleotides were complementary to nucleotides at positions 25–50 of ${\text{tRNA}}_{\text{cmnm}5\text{s}2\text{UUG}}^{\text{Gln}}$ and at positions 25–49 of ${\text{tRNA}}_{\text{GGG}}^{\text{Pro}}$. Northern blots were visualized by Phosphor-Imager analysis (S3 Fig).

### Purification of FLAG-proteins

The Flag-tagged proteins, expressed in strain LMG194, were purified essentially as described by Moukadiri *et al* [36] except that the induced cells were grown over night at 30°C. The respective *mnmH* genes were amplified by PCR using FLAG-specific *mnmH* primers and cloned into the expression vector (pBAD TOPO TA) to generate the three FLAG-MnmH derivatives. The proteins were purified by standard procedure and routinely stored at -20°C in the presence of 15% glycerol.

### Determining the modification pattern in tRNA bound to purified MnmH proteins

Fractions of purified protein were pooled, mixed with equal volume of phenol, and the nucleic acid was precipitated from the water phase by 2.5 volumes of ethanol with 1% KAc added. The precipitate was dissolved in water and once more the tRNA was extracted with phenol and the tRNA was precipitated with 2.5 volume of ethanol, washed with 70% ethanol, dissolved in 100 μl MQ-water, degraded into nucleosides as described above, and the degraded products were analysed by HPLC. The modification pattern was compared to digested total tRNA from *E*. *coli*.

## Results

### The size and not the chemical features of amino acids at position 67 mediates increased geranylation activity of tRNA *in vivo*

Four independent +1 frameshift suppressor mutants were obtained earlier [5–7], which all had alterations at position 67 of MnmH located in the conserved 36 amino acid insertion in the rhodanese domain, which is found in many MnmH orthologues (Fig 2). Although the alterations were either a basic (Arg) or an acid amino acid (Glu), it still suggested that features at this position might be critical to obtain an increased level of geranylation of tRNA. To get more information about this aspect, we randomly scrambled the three nucleotides of codon 67 of *mnmH* on a plasmid. To analyze the geranylation activity of these altered MnmH proteins under the same condition as the original mutants, we transferred the mutated *mnmH* genes to the chromosome. We obtained nine new alterations at position 67 of MnmH inducing a +1 frameshift and concomitantly increasing the synthesis of (c)mnm^5^ges^2^U34 in tRNA (Table 1). The chemical properties of the amino acid side chain were all different (acid, basic hydrophobic, hydrophilic) but a common feature of the alterations was that the side chains have a larger van der Waals volume [37] than glycine, which is the amino acid in position 67 of the wild type MnmH (Table 1). Therefore, it appears that it is a space alteration in the vicinity of position 67 that is critical to generate an increased level of (c)mnm^5^ges^2^U34 in tRNA and thereby an ability to suppress the +1 frameshift mutation in the *his*-operon. Apparently, space alterations at or in the vicinity of position 67 changes the structure of the MnmH and thereby increases the endogenous weak activity of wild type MnmH to synthesize (c)mnm^5^ges^2^U34 in tRNA. We also considered that the 36 amino acid insertion might act as a negative element blocking an activity to generate (c)mnm^5^ges^2^U34. However, an MnmH protein with these 36 amino acids deleted, did not mediate any formation of (c)mnm^5^ges^2^U34 (data not shown).

**Table 1:** Size and not the chemical feature of the amino acid side chain at position 67 is critical for increased geranylation activity of MnmH. ND, not detected. Relevant genotype: Besides indicated alterations of MnmH at position 67 encoded from the relevant *mnmH* gene located on the chromosome, all strains contain *zbb-2558*::Tn10*d*Tc (close to *mnmH*), *hisO1242*, *hisD10122* (CCC-CAA; +1 frameshift mutation), *zdd-2532*::*Cat*. Frameshifting (FS) of the indicated strains were monitored as growth of single cells on plates without histidine after 4 days at 37°C (+++, large colonies; ++, medium size colonies, +, small size colonies and (+), very small colonies). Wild type shows no growth under the same conditions. The frameshift phenotype is caused by shortage of Gln-${\text{tRNA}}_{\text{cmnm}5\text{s}2\text{UUG}}^{\text{Gln}}$ (cmnm^5^s^2^U) due to the formation of cmnm^5^ges^2^U34 of this tRNA. This inhibition of the glutaminylation reaction causes lower levels of charged non-geranylated Gln-${\text{tRNA}}_{\text{cmnm}5\text{s}2\text{UUG}}^{\text{Gln}}$ which in turn induces the frameshifting activity [10]. Geranylated compounds were determined by HPLC analysis of tRNA from the indicated strains.

Strain	aa at pos.67	Chemistry of aa side chain	V	Total ges^2^-derivatives/Ѱ (x10^-3^)^b^	FS
GT7321	G (wt)	Hydrophobic	63.8	ND	-
GT8216	E	Acidic	140.8	17 ± 8.2	+
GT8187	I	Aliphatic/hydrophobic	164.9	13 ± 6.7	+++
GT8188	T	Weak acidic	120.0	19 ± 4.3	+++
GT8189	L	Aliphatic/hydrophobic.	164.6	15 ± 1.5	++
GT8190	R	Strongly basic	192.8	11 ± 0.9	++
GT8191	N	-	127.5	10 ± 3.2	(+)
GT8192	C	Hydrophobic/acidic	103.5	16 ± 2.5	+
GT8193	H	Aromatic/weak basic	159.3	18 ± 6.7	+
GT8194	V	Aliphatic/hydrophobic	139.1	15 ± 1.4	+
GT8195	M	Hydrophobic	167.7	23 ± 1.0	+

### Alterations in or close to the conserved 36 amino acid insertion in the rhodanese domain increases the level of (c)mnm^5^ges^2^U34 in tRNA

**Fig 3:**
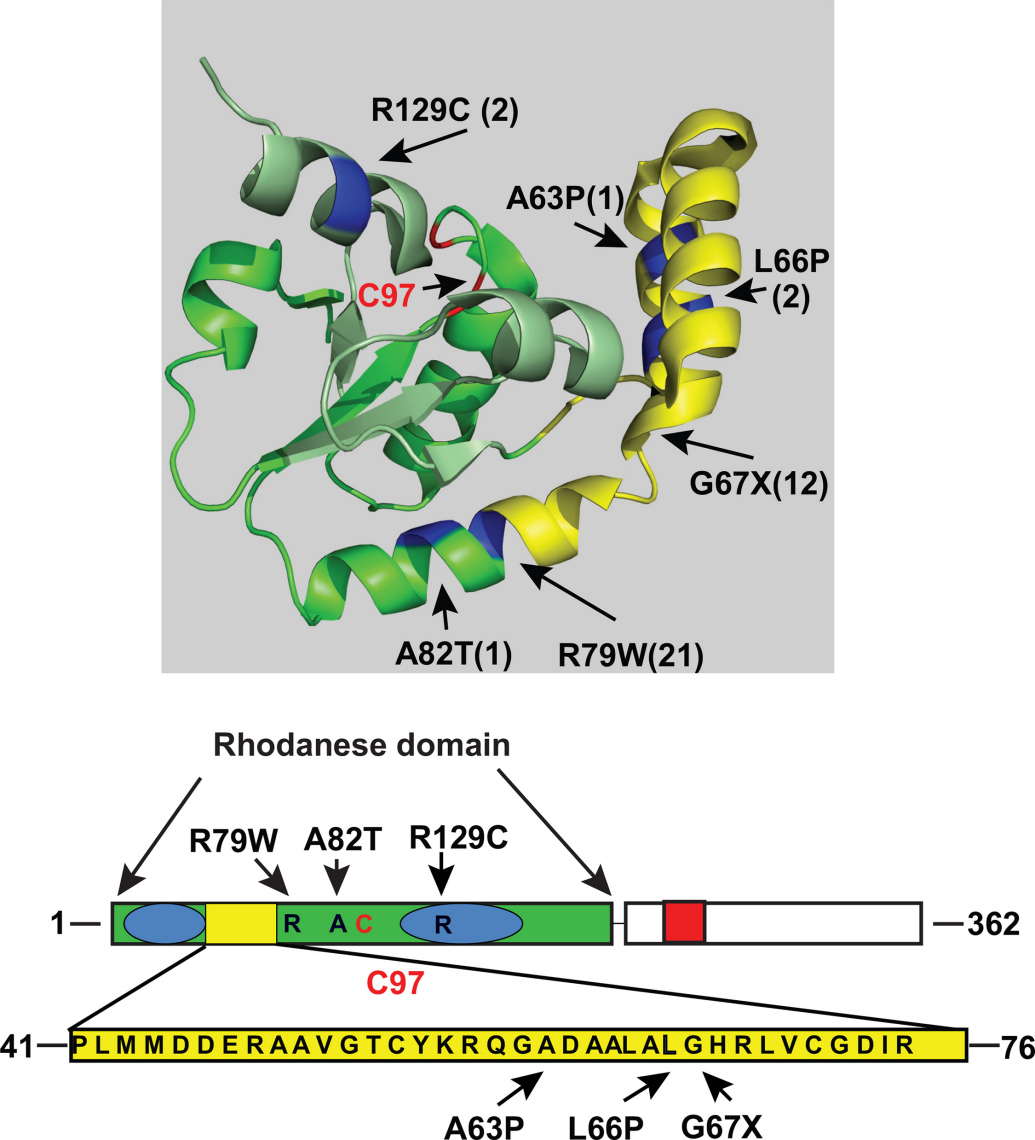
Distribution of the 39 independent amino acid substitutions in MnmH that generate increased geranylation activity. Thirty-five (35) of these had amino acid substitutions in or in close proximity to the conserved 36 amino acid insertion in the rhodanese domain present in MnmH orthologues. X at position 67 indicates the various alterations were obtained in this position as shown in Table 1.

We have isolated earlier one mutant with increased geranylation activity following a genome wide selection of frameshift suppressors [7], and three mutants were isolated 1970 as +1 frameshift suppressors by Riddle and Roth [5, 6] (Table 2). Thus, four independently isolated mutants have been characterized and all were located in the rhodanese domain suggesting that this domain is pivotal for increasing the geranylation activity. To further characterize the requirement for increased geranylation activity, we randomly mutagenized the region containing the *mnmH* gene and selected clones with increased +1 frameshift activity and accordingly an increased geranylation of ${\text{tRNA}}_{\text{cmnm}5\text{s}2\text{UUG}}^{\text{Gln}}$ (For details see Materials and Methods). Since the selection procedure used required enhanced ability to suppress a +1 frameshift mutation, we did not expect any mutants with a decreased geranylation activity. In total we characterized 39 independently isolated *mnmH* mutants, of which 35 were obtained by the above described localized mutagenesis. Of the latter, four were double mutants (two alterations at positions 69 and 66 in conjunction with alteration in position 67 and one (position 54) in conjunction with an alteration R79W). Thirty five of the 39 mutants had alterations in or in the vicinity of the conserved 36 amino acid insertion in the rhodanese domain (Fig 3). None of the mutants had an alteration in the P-loop domain, suggesting that potential alterations in this domain are not able to increase the geranylation activity. The results suggest that important features to increase the geranylation activity reside in the rhodanese domain.

**Table 2:** Characterization of 39 independent isolated mutants with increased frameshifting activity caused by increased geranylation activity following localized mutagenesis of the *mnmH* gene or by whole genome selection for increased frameshifting activity. **A.** Mutants were isolated following localized mutagenesis as described in M-M. All alterations in MnmH were obtained by the mutagen hydroxylamine except the alterations A63P and one of the two L66P alterations which were obtained using overexpression of *dinB* and the mutagen nitrosoguanine, respectively. In addition three mutants (One G67E and two G67R) obtained by Riddle and Roth [6] and one mutant (G67R) obtained by Jäger *et al* [7] are also included in the table. All these mutants have increased +1 frameshifting activity (data not shown), which is a caused by increased geranylation of tRNA. We did not observe a simple correlation between ability to suppress a +1 frameshift mutation to the level of cmnm^5^ges^2^UpU, which might not be expect, since induction of frameshifting is complex although partly dependent on a decreased level of charged Gln-${\text{tRNA}}_{\text{cmnm}5\text{s}2\text{UUG}}^{\text{Gln}}$ [4]. B. Total ges^2^-derivatives consist of the level of cmnm^5^ges^2^U and mnm^5^ges^2^U determined as described in S1 Fig. Standard deviations are given as +/-. C. Not done. The level of the geranylation in these double mutants was not determined since they all had an alteration, which as a single alteration give increased geranylation activity. They all had increased frameshifting activity. D. Values are from Table 1. E. Following independent isolated mutants with G67R alteration in MnmH are present in strains GT945 and GT946 [6]. In addition, but not included in this table, are strains GT8190 (G67R) and GT8194 (G67V), which were obtained by scrambling the codon in position 67 (Table 1).

Alterations in MnmH ^a)^	No. of mutants	Total ges^2^-derivatives/ Ѱ (x10^-3^)^b)^
Wild (88type(GT7321	-	<1
G67E^d^	2	17 ± 8.2
R129C	2	9 ± 4.7
G67E, R69Q	1	Not done^c^
R79W	21	16 ± 3.5
A63P	1	15 ±6.5
L66P	2	17 ± 4.8
A82T	1	14 ± 6.9
R79W, C54C	1	Not done^c^
G67R, L66L	2	Not done^c^
G67R^e)^	5	11 ± 0.9
G67V^d^	1	15 ±1.4

Of the amino acid substitutions obtained in MnmH increasing the level of geranylation of tRNA, three were in 100% conserved positions (A63, G67 and R129), one in a 86% conserved position (L66), one in a 71% conserved position (R79), and one in a 50% conserved position [9]. Thus, alterations that increase the geranylation activity were all in the conserved or semiconserved positions of the rhodanese domain.

### High level of geranylated ${\text{tRNA}}_{\text{cmnm}5\text{s}2\text{UUG}}^{\text{Gln}}$, ${\text{tRNA}}_{\text{mnm}5\text{s}2\text{UUU}}^{\text{Lys}}$, and ${\text{tRNA}}_{\text{mnm}5\text{s}2\text{UUC}}^{\text{Glu}}$ does not grossly affect cellular growth

The three tRNAs having the (c)mnm^5^s^2^U derivatives are about 50–70% geranylated in a strain having the MnmH(G67E) amino acid substitution (Table 3). One would then expect a reduction in growth unless the geranylated tRNA is able to efficiently decode the message. The charging level of ${\text{tRNA}}_{\text{cmnm}5\text{s}2\text{UUG}}^{\text{Gln}}$ is reduced to 26% as compared to the wild type level of 86% [4], a reduction that also results in increased frameshifting [4]. However, the level of charging of ${\text{tRNA}}_{\text{mnm}5\text{s}2\text{UUU}}^{\text{Lys}}$ and ${\text{tRNA}}_{\text{mnm}5\text{s}2\text{UUC}}^{\text{Glu}}$ is not effected [8]. Still, the geranylation of ${\text{tRNA}}_{\text{mnm}5\text{s}2\text{UUU}}^{\text{Lys}}$ induces +1 and -1 frameshifting and the efficiency of reading the glutamic acid codon GAA is reduced by 50% due to the geranylation of ${\text{tRNA}}_{\text{mnm}5\text{s}2\text{UUC}}^{\text{Glu}}$ [8]. All these observed phenotypes would suggest that the geranylation may reduce the growth. We therefore tested the influence on growth of three strains [GT8277 (*ΔmnmH*), GT5688 (*mnmH*^*+*^), and strain GT5687 (*mnmH204* (G67E)] with no, low level, and high level of geranylated tRNA, respectively. However, there was no difference in growth, measured as size of single cell colonies, between these strains on three different growth media (Rich medium, glucose and acetate minimal medium) and at three different temperatures (30, 37 and 41°C). Also, no difference in the specific growth rate constant was observed between these strains in rich liquid medium at 37°C (S2 Fig).

**Table 3:** The altered MnmH (G67E) mediates high level of geranylation of the tRNAs specific for Gln, Lys and Glu. A. The indicated levels of geranylated derivatives were calculated by assuming that the reduced level of (c)mnm^5^s^2^U has been converted to the corresponding geranylated derivative. B. Analysis of the purified species ${\text{tRNA}}_{\text{cmnm}5\text{s}2\text{UUG}}^{\text{Gln}}$. **C.** HPLC analysis of bulk tRNA from strain GT6680 (*ΔmnmH*) revealed no geranylated tRNA (unpublished observation). D. The level of geranylated derivatives from an *E*. *coli* (*ΔmnmH*) strain containing a plasmid harboring an *mnmH* (G67E) allele.

	*mnmH*^*+*^	*ΔmnmH*	*mnmH204*(G67E)	
	% ges^2^-derivatives of total (c)mnm^5^s^2^U^a)^	% ges^2^-derivatives of total (c)mnm^5^s^2^U ^a)^	% total ges^2^-derivatives of total (c)mnm^5^s^2^U ^a)^	
${\text{tRNA}}_{\text{cmnm}5\text{s}2\text{UUG}}^{\text{Gln}}$ ^b)^	< 3	Not done	70	[4]
In tRNA Gln, Lys, and Glu	8	Not detected ^c)^	50	[4]
In tRNA Gln, Lys and Glu.	3–7	Not detected	45 ^d)^	[8]

### The hydrophobic geranyl group does not cause an enrichment of geranylated tRNA in the outer or inner membranes

The envelope of a gram negative bacteria consists of an inner and an outer membrane with a peptidoglycan cell wall and a periplasmic space in between. Whereas the outer membrane consists of lipopolysaccharides and proteins the inner membrane consists of a bilayer with a hydrophobic layer of fatty acids. A human tRNA^Sec^ containing the prenylated modified nucleoside 6-isopentenyl-adenosine (i^6^A) has an anticodon loop that is highly hydrophobic. Such tRNA is retained in the hydrophobic HeLa cell membrane demonstrating that some tRNAs may be located in another cellular compartment than the cytoplasm [38]. The prenylated geranyl group of (c)mnm^5^ges^2^U, which is also highly hydrophobic, might mediate insertion of a geranylated tRNA into the hydrophobic lipid bilayer similar to the insertion of tRNA^Sec^ into the human membrane. Thus, geranylation of tRNA might thereby reduce the concentration of such tRNA in the cytoplasm and hence reduce the amount of ternary complex available for translation. Shortage of ternary complex may induce frameshifting by inducing a pause of ribosome waiting for the ternary complex to bind to the ribosomal A-site and such an event will induce a frameshifting event by the peptidyl-tRNA [2, 7, 39] as is observed for geranylated Gln-tRNA [4] and geranylated Lys-tRNA [8]. Moreover, such shortage of the ternary complex may also reduce the rate of translation as was observed for GAA(Glu) codon [8].Thus, it was of interest to investigate if the hydrophobic geranyl group would mediate an enrichment of geranylated tRNA to the membranes and, if so, explain some of the above mentioned phenotypes. We therefore prepared outer and inner membranes and monitored the presence of ${\text{tRNA}}_{\text{cmnm}5\text{s}2\text{UUG}}^{\text{Gln}}$, which potentially might be geranylated, and ${\text{tRNA}}_{\text{GGG}}^{\text{Pro}}$, which is not a substrate for geranylation, in the membrane fractions. We used ^32^P-labelled oligonucleotides specific for these tRNAs as probes in a Northern blot analysis of membranes of three strains [GT8277 (*ΔmnmH*), GT5688 (*mnmH*^*+*^), and strain GT5687 (*mnmH204* (G67E)] with no, low level, and high level of geranylated tRNA, respectively. No positive signal for ${\text{tRNA}}_{\text{cmnm}5\text{s}2\text{UUG}}^{\text{Gln}}$ or for ${\text{tRNA}}_{\text{GGG}}^{\text{Pro}}$ was observed in the outer membrane fraction, whereas in the inner membrane fraction signals for ${\text{tRNA}}_{\text{cmnm}5\text{s}2\text{UUG}}^{\text{Gln}}$ and ${\text{tRNA}}_{\text{GGG}}^{\text{Pro}}$ were observed in all three strains (S3 Fig). Thus, apparently at least these two tRNAs were associated with the inner membrane. However, the signal for the presence of ${\text{tRNA}}_{\text{cmnm}5\text{s}2\text{UUG}}^{\text{Gln}}$ was similar in these three strains demonstrating that geranylation of the tRNA did not enrich the association of this tRNA with the inner membrane. We conclude that geranylation of tRNA does not induce an enrichment of tRNA associated with the membranes.

### An intact Walker motif in the P-loop domain, but not an intact catalytic cysteine in the rhodanese domain, is required for the geranylation activity

As pointed out above, the rhodanese domain is critical for the enhancement of the geranylation activity, since all alterations of MnmH obtaining this feature resided in this domain. Therefore, the catalytic cysteine (C97) in this domain might be required for this activity in a way similar to the importance of it in selenation [9]. The functionality of C97 in geranylation was tested by constructing amino acid substitutions C97A and C97S. Although the non-conserved cysteine at position 96 is not required for selenation [9], we also tested its relevance for the geranylation reaction due to its close location to the catalytic cysteine in the rhodanese domain. The geranyl group is transferred *in vitro* from geranylpyrophosphate (GePP) by MnmH to the tRNA(see below). The P-loop domain contains a Walker motif (GCTGSK_155_T, Fig 2), which is a potential nucleotide binding site and therefore a potential binding site for GePP. We therefore tested the relevance of K155 in this sequence for geranylation by altering K155 to A155. These alterations were combined with the G67E amino acid substitution in MnmH, which mediates increased geranylation activity as compared to wild type MnmH. Analysis of mutants having the corresponding mutations on the chromosome demonstrated that the Walker motif in the P-loop domain must be intact to obtain geranylation activity whereas altering C97 or C96 reduced the level of geranylation but did not abolish it (Table 4). To increase the geranylation activity we placed the *mnmH* gene and its derivatives on a plasmid. In this case the K155A alteration still reduced substantially the activity of the MnmH^+^ as well as of the MnmH(G67E) derivative (Table 4). The alterations of C97 (either to an Ala or to a Ser) or C96S reduced the activity but much less than the K155A amino acid substitution. Thus, the C97 or C96 is not absolutely required for geranylation although a structural change in the neighborhood of them caused by changing the Cys to either Ser or Ala reduced the activity. We conclude that the catalytic cysteine in the rhodanese domain is not required for geranylation in sharp contrast to the absolute requirement of C97 for selenation, whereas an intact Walker motif in the P-loop domain is. This latter result implies that the sequence GCTGSK_155_T in the P-loop domain may be the GePP binding site.

**Table 4:** Intact cysteines at positions 97 or 96 are not absolutely required for geranylation of tRNA whereas intact P-loop is.

Strain	mnmH mutations located on the chromosome	Overexpressing MnmH derivatives from plasmid	Total ges^2^-derivatives/Ѱ
GT7321	*mnmH*^*+*^	-	< 0.001(<1%)
GT1484	*mnmH204*(G67E)	-	0.069 (100%)
GT8243	*mnmH182*(G67E,K155A) K155A	-	<0.001 (<1%)
GT8287	*mnmH221* (G67E, C97S)	-	0.060 (87%)
GT8286	*mnmH222*(G67E, C96S)	-	0.045 (65%)
GRB2407	*ΔmnmH*	pUST317 (*mnmH*^*+*^)	0.050 (100%)
GRB2408	*ΔmnmH*	pUST318 (*mnmH4*, K155A)	0.003 (6%)
GRB2409	*ΔmnmH*	pUST319 (*mnmH5*, C97A)	0.030 (60%)
GRB2405	*ΔmnmH*	pUST315 (*mnmH204*, G67E)	0.345 (100%)
GRB2406	*ΔmnmH*	pUST 316 (*mnmH182*, G67E, K155A)	0.021 (6%)
GRB2410	*ΔmnmH*	pUST320 (*mnmH223*, G67E, C97A)	0.089 (26%)
GRB2419	*ΔmnmH*	pUST321(*mnmH221*,G67E, C97S)	0.118 (34%)

### The accumulation of geranylated derivatives *in vivo* is partly sensitive to the presence or absence of the (c)mnm^5^-side chain of the target nucleoside (c)mnm^5^s^2^U34

The first step in the synthesis of the cmnm^5^-side chain is catalyzed by the MnmEG complex. In the presence of Gly the cmnm^5^-side chain is first synthesized and this side chain is thereafter cleaved by the MnmC(o) activity to form the nm^5^-side chain. When instead ammonium ion is present the MnmEG complex synthesizes directly the nm^5^-side chain. To generate the mnm^5^-side chain the nm^5^-side chain formed by either of these reactions is methylated by MnmC(m) (Fig 1). In the wild type, the cmnm^5^-side chain is present only in the ${\text{tRNA}}_{\text{cmnm}5\text{s}2\text{UUG}}^{\text{Gln}}$, whereas the ${\text{tRNA}}_{\text{mnm}5\text{s}2\text{UUU}}^{\text{Lys}}$ and ${\text{tRNA}}_{\text{mnm}5\text{s}2\text{UUC}}^{\text{Glu}}$ contain the mnm^5^-side chain. In the presence of Gly, deletion of the *mnmC* gene (*ΔmnmC*) results in the accumulation of the cmnm^5^-side chain in the Gln-, Lys, and Glu-tRNAs. Deleting the *mnmE* gene results in no synthesis of a side chain and s^2^U accumulates in all these three tRNAs. To test the influence of the (c)mnm^5^-group on the geranylation activity, we used strains with the *mnmH204* (G67E) mutation in conjunction with either *ΔmnmE* or *ΔmnmC* deletion and monitored thereby the level of geranylation of tRNA with no side or the cmnm^5^- side chain, respectively, at position 5 of the target nucleoside (c)mnm^5^s^2^U. Table 5 shows that in all strains with different side chains at position 5 of the target nucleoside, the level of ges^2^U-derivatives were reduced by about 50% suggesting that the geranylation activity is only partly sensitive to the length or absence of the (c)mnm^5^-side chain.

**Table 5:** Geranylation *in vivo* is only partly sensitive to the length or absence of the side. Transfer RNA from strains with indicated alterations on the chromosome was prepared and the total level of geranylated derivatives are given (+/-, standard deviation). In wild type background cmnm^5^s^2^U and mnm^5^s^2^U are present. tRNA of a *ΔmnmC* and a *ΔmnmE* mutant contains cmnm^5^s^2^U and s^2^U, respectively, prior to their geranylation.

Strains	ges^2^-derivatives/Ψ in tRNA from strains with indicated genetic background				
	Pos. 67	Other pos.	*mnmCE*^*+*^(wt) [(c)mnm^5^ges^2^U]	*ΔmnmC* [cmnm^5^ges^2^U]	*ΔmnmE*
					[ges^2^U]
GT7321	wt	wt	<0.001		
GT1484	G67E	wt	0.040 ± 0.008 (100%)		
GT8243	G67E	K155A	<0.001		
GT6680	*ΔmnmH*		<0.001		
GT8270	G67E	K155A		<0.001	
GT8253	*ΔmnmH*			<0.001	
GT8269	G67E	wt		0.014 ± 0.002 (35%)	
GT8271	G67E	wt			0.016 **±** 0.003 (40%)
GT8272	*ΔmnmH*				<0.001

### Purified MnmH(G67E) and MnmH(G67E, K155A) but not MnmH^+^ bind the chaperon GroEL

**Fig 4:**
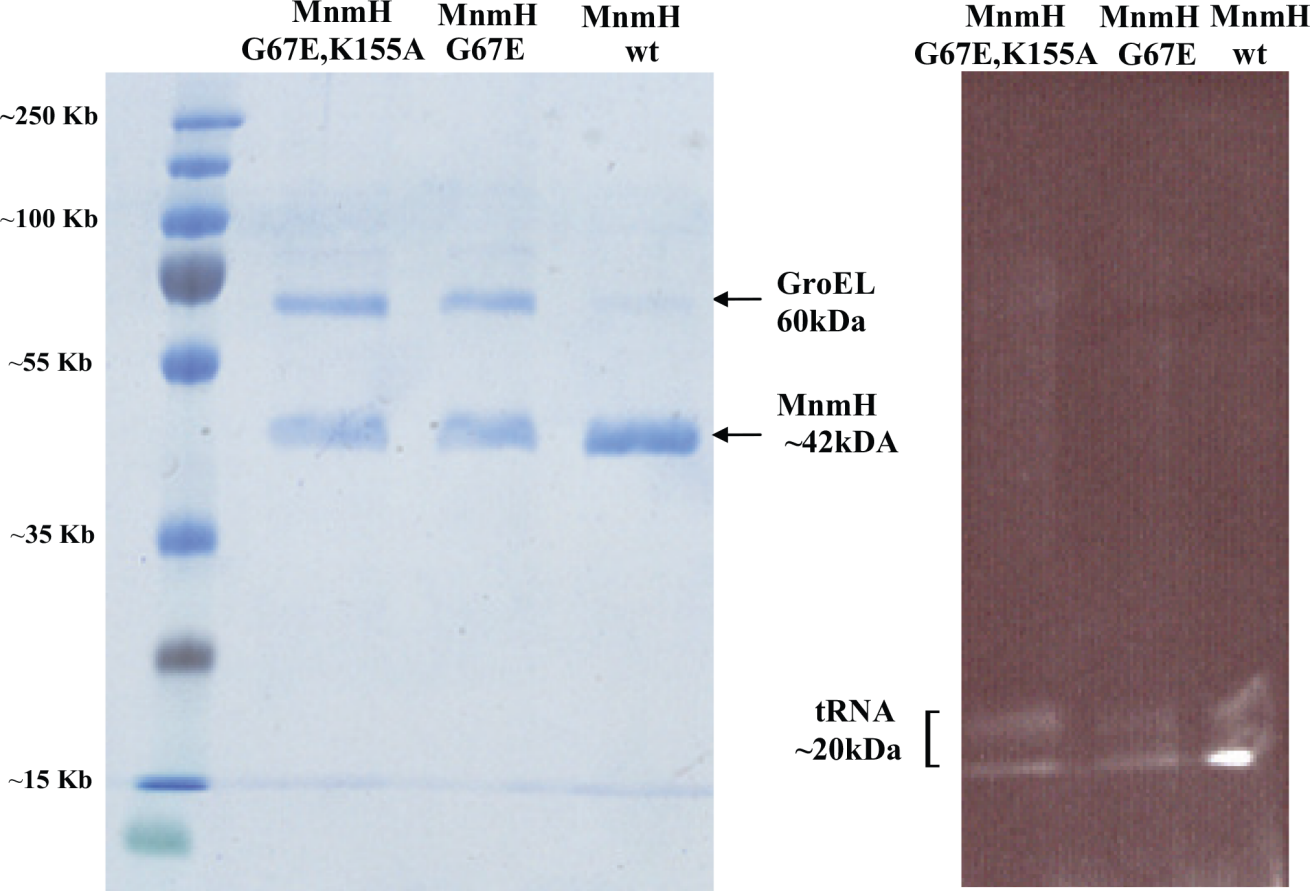
Characterization of purified MnmH from the double mutant G67E K155A and the G67E mutant and the wild type (*mnmH*^*+*^). The corresponding MnmH FLAG- derivatives were expressed in strain LMG194, which is *mnmH*^*+*^. SDS-PAGE of the different Flag-MnmH proteins (about 7 μg) stained by coomassie blue to detect proteins (**A**) and stained with ethidium bromide to detect nucleic acid (**B**). The protein band above MnmH is GroEL as determined by amino acid sequencing. GroEL is also present as a weak band in wild type. All preparations also contain tRNA and apparently the wild type MnmH contains more of tRNA than the two mutant forms of MnmH.

To characterize the geranylation further, we purified the FLAG-tagged MnmH proteins expressed from three different plasmids–the wild type MnmH^+^, the MnmH(G67E) mutant, and the double mutant MnmH(G67E, K155A). Fig 4 shows that all preparations contain, besides the MnmH proteins, GroEL and tRNA (the GroEL was identified by N-terminal amino acid sequencing). The mutant forms contain substantial amount of GroEL whereas only a weak band of GroEL was observed in the wild type preparation. GroEL is a chaperon suggesting that the aberrant proteins MnmH(G67E) and MnmH(G67E, K155A) require this chaperon to be folded properly. Presence of such aberrant proteins might also induce synthesis of GroEL but this was not the case, since the level of GroEL as monitored by GroEL specific antibodies was the same in the strain overproducing these proteins as in wild type strain without any plasmid (data not shown). We also tried to purify other mutant forms of MnmH but such preparations had degraded MnmH and very low amount of intact peptide in conjunction with large amount of GroEL (data not shown). We judged that these preparations were not suitable to test the *in vitro* geranylation activity.

### The purified wild type MnmH and the MnmH(G67E) mutants but not the double mutant MnmH(G67E K155A) transfer *in vitro* a geranyl group from geranylpyrophosphate to tRNA

**Fig 5:**
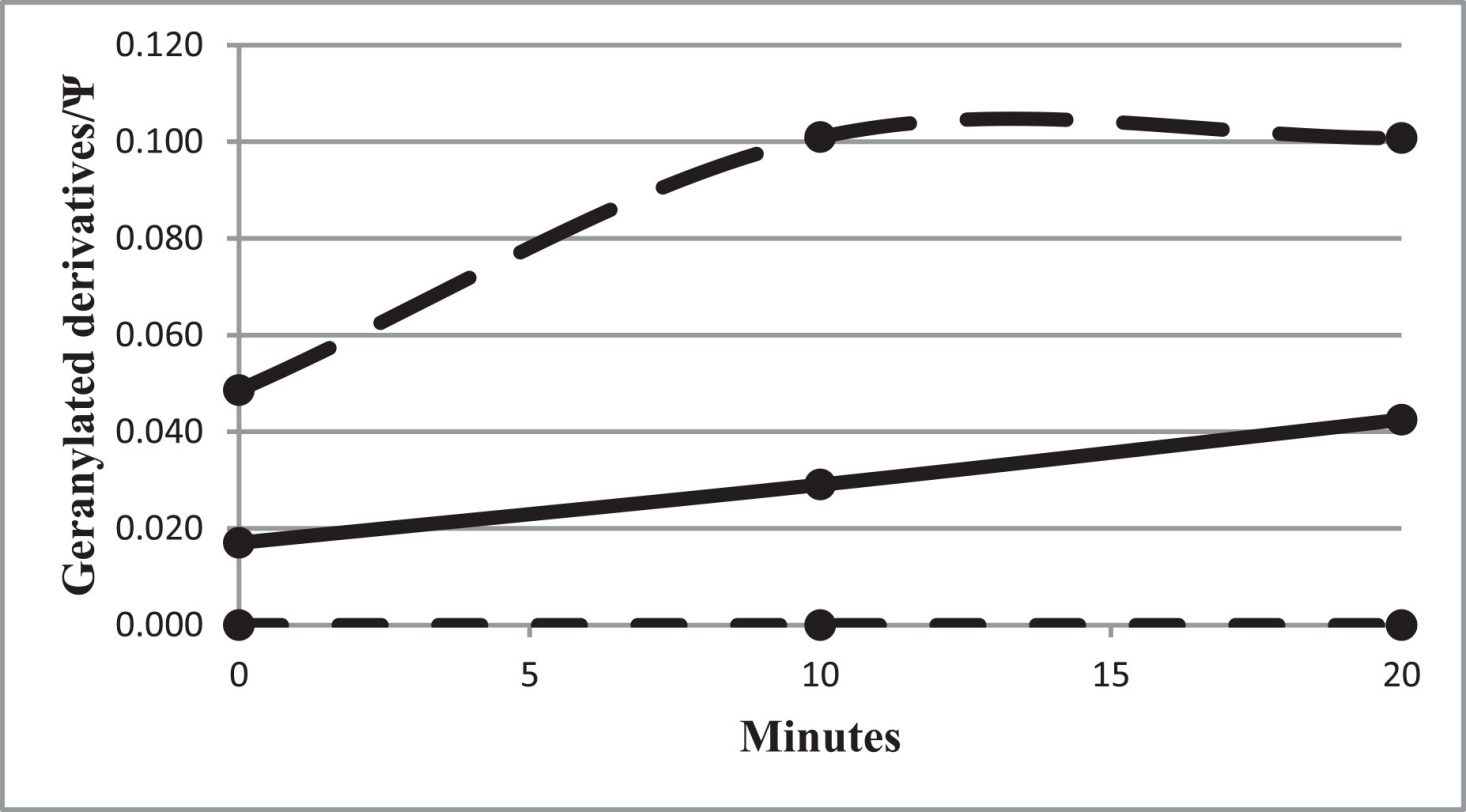
*In vitro* geranylation of *ΔmnmH* tRNA, which is devoid of geranylated (c)mnm^5^s^2^U derivatives. MnmH (G67E) (dashed line), MnmH+ (solid line) and double mutant MnmH (G67E, K155A) (narrow dashed line) proteins were used as enzyme source. Corrected for the presence of GroEL in the different enzyme preparations, the specific activities (min^-1^, ug MnmH protein^-1^) were 0.12x 10^−3^ (wild type), 0.40 x 10^−3^ (G67E), and 0 (G67E, K155A). The reaction was stopped at indicated times by phenol extraction and the geranylated compounds in the reaction mixture were determined by HPLC after digestion with P1 as described in Material and Methods. Note, that the zero time points for MnmH (G67E) and MnmH^+^ already show the presence of geranylated compound(s) whereas this is not the case for the double mutant MnmH (G67E, K155A).

To characterize further the geranylation activity of some of the altered MnmH, we tested purified MnmH proteins for their geranylation activity *in vitro*. Transfer RNA was purified from strain GT6680 (*ΔmnmH*), since such tRNA does not contain any (c)mnm^5^ges^2^U derivatives and such ges^2^-deficient tRNA should be optimal as an acceptor for a potential transfer of a geranyl (ge) group from the geranyl group donor GePP to tRNA. Since an intact P-loop is required *in vivo* to generate (c)mnm^5^ges^2^U34, the double mutant (G67E, K155A) should not possess any geranylation activity. We also expected no or only weak activity for the wild type form of the enzyme whereas the MnmH(G67E) enzyme should be the most active enzyme to geranylate the tRNA. Indeed, the MnmH(G67E) catalyzed the formation of the mnm^5^ges^2^U34 in tRNA in a time dependent manner (Fig 5). The reaction was also enzyme dependent (data not shown). The wild type MnmH^+^ enzyme was also able to transfer a geranyl group *in vitro* whereas the double mutant MnmH(G67E,K155A) was inactive as was expected from the analysis *in vivo* of the altered Walker motif (Table 3). Note, that the zero time point for the wild type and the MnmH(G67E) enzymes had a significant level of geranylated tRNA, which apparently did not require any incubation to be formed. The inactive MnmH double mutant (G67E, K155A) had no geranylated tRNA at zero time. Apparently, the tRNA bound to this enzyme (Fig 4) is not geranylated.

### The purified MnmH proteins have a bound tRNA population that is enriched by the geranylated form of the tRNA

**Fig 6:**
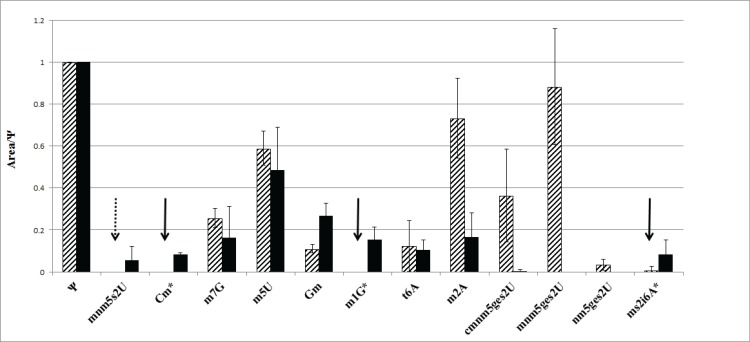
HPLC analysis of tRNA bound to purified MnmH^+^ enzymes (hatched bars) and cytoplasmic tRNA from wild type strain (black bars). All figures are relative to the level of Ψ. Nucleosides denoted * are not present in the Gln-, Lys, and Glu-tRNA, which are the only tRNAs being substrates for geranylation. Thus, the * denoted nucleosides, which should not be present in the bound tRNA if this is enriched by the geranylated tRNA. Whereas ${\text{tRNA}}_{\text{mnm}5\text{s}2\text{UUC}}^{\text{Glu}}$ contains in position 37 an unmodified A37, ${\text{tRNA}}_{\text{mnm}5\text{s}2\text{UUU}}^{\text{Lys}}$ and ${\text{tRNA}}_{\text{cmnm}5\text{s}2\text{UUG}}^{\text{Gln}}$ contain t^6^A37 and m^2^A37, respectively, in this position. Therefore, these modified nucleosides are expected to be present in the geranylated tRNAs and therefore enriched in the bound tRNA, which is the case. Note, that (c)mnm^5^s^2^U is not detected (denoted with a dashed arrow) in the enzyme bound tRNA suggesting that this nucleoside, which is normally present in the three tRNA species being a substrate for geranylation, is completely converted to its geranylated derivatives in the tRNA bound to the enzyme. An arrow (↓) in the other cases denotes that the indicated derivatives were not detected (or present at a very low amount–ms^2^i^6^A) and in all these cases are not expected provided the MnmH protein is enriched for the three tRNA species (Gln, Lys and Glu).

In the wild type MnmH preparation a strong band of tRNA (Fig 4) was observed consistent with the observation by Wolfe *et al*. [9], C:\GetARef\Refs\Refsmanus\MnmH-2014.ref #15; who estimated that two moles of tRNAs were bound per mole of wild type MnmH^+^ [9]. Transfer RNA was also bound to the MnmH(G67E) and MnmH (G67E,K155A) mutant forms of MnmH (Fig 4). As mentioned above we also noticed that monitoring geranylation activity *in vitro*, samples taken at zero time of incubation had a substantial level of geranylated tRNA (see Fig 5). This might indicate that the purified enzymes had geranylated tRNA bound to it. To test this, we prepared tRNA from enzyme preparation of wild type MnmH^+^ as well as from some of the mutant forms of MnmH and analyzed the modification pattern of the enzyme bound tRNA. Modified nucleosides (Cm, m^1^G, and ms^2^i^6^A) not present in tRNAs specific for Gln-, Lys, or Glu-tRNA, which are the only substrates for geranylation, were underrepresented compared to bulk tRNA (indicated by arrows in Fig 6). On the other hand modified nucleosides present in the Gln-Lys- and Glu- tRNAs (t^6^A, m^2^A, (c)mnm^5^ges^2^U) were overrepresented. Similar results were obtained when tRNA bound to MnmH(G67E) enzyme was analyzed (data not shown). We conclude that tRNAs bound to the enzymes were enriched with the geranylated derivatives. Apparently, the MnmH proteins have strong affinity for such tRNA (Fig 6).

## Discussion

The first alteration in MnmH was discovered in a mutant mediating the ability to suppress +1 frameshift mutation and this induced phenotype is due to geranylation of ${\text{tRNA}}_{\text{cmnm}5\text{s}2\text{UUG}}^{\text{Gln}}$ [4] alteration is within the 36 amino acid insertion in the rhodanese domain conserved among bacterial and archaeal MnmH orthologues (Fig 2). To obtain more information what other changes in the MnmH protein would induce a similar phenotype we isolated several independent mutants with increased suppressor activity and accordingly an increased level of geranylation of the tRNA. Interestingly, the alterations in position 67, in which the first isolated mutants were altered, suggest that it is not the chemical features but the size of the amino acid that is important for the induced phenotype (Table 1). However, isolating randomly induced mutations in the *mnmH* gene we found alterations outside this conserved 36 amino acid insertion but they were all in the rhodanese domain (Table 2, Fig 3). Still, the alterations were in the vicinity of this 36 amino acid insertion and all were close to the region where the catalytic Cys97 of the rhodanese domain is located. Thus, the rhodanese domain is important for the geranylation activity. This is also the case for the selenation activity, since the latter has an absolute requirement for an intact Cys97 whereas the geranylation activity does not (Table 3). The two activities are mechanistically different although the features of the rhodanese domain are important for both. We also show that the Walker motif in the P-loop domain is required for geranylation implying that this sequence might be important for the binding of GePP and this would argue against that MnmH is a moonlighting protein. Even though this sequence is outside the rhodanese domain the importance of an intact P-loop domain points towards a role in the geranylation reaction may be through binding of GePP. Wolfe et al [9] suggested that the P-loop domain binds tRNA and thus is required for both activities. This suggestion is supported by analysis of a MnmH orthologue in Archaea. In these organisms the two domains of MnmH orthologue are encoded by two genes of which the P-loop domain is encoded by a gene upstream of the gene encoded the rhodanese domain. Still, both half of the MnmH protein have to be present to obtain selenation activity and tRNA binding [17].

The MnmH (G67E) mutant was discovered as being +1 frameshift suppressor [4]. Since this alteration of MnmH also resulted in reduced charging of ${\text{tRNA}}_{\text{cmnm}5\text{s}2\text{UUG}}^{\text{Gln}}$ this suppressor phenotype is consistent with the “peptidyl slippage model” [7, 38]. This model suggests that a shortage of an aminoacylated tRNA reduces the rate of entry to the ribosomal A-site by the corresponding ternary complex (aa-tRNA*GTP*elongation factor Tu (EF-Tu)). Such a shortage will induce a ribosomal pause and thereby increases the probability of a slippage one nucleotide forward by the peptidyl-tRNA. Thus, the fact that geranylation of the ${\text{tRNA}}_{\text{cmnm}5\text{s}2\text{UUG}}^{\text{Gln}}$ reduces the charging of ${\text{tRNA}}_{\text{cmnm}5\text{s}2\text{UUG}}^{\text{Gln}}$ explains the frameshift suppressor phenotype of the MnmH (G67E) mutant [4]. According to Dumelin *et al* [8] geranylation of ${\text{tRNA}}_{\text{mnm}5\text{s}2\text{UUU}}^{\text{Lys}}$ is fully charged but still induces +1 frameshift errors, which is not consistent with the proposed peptidyl-tRNA slippage model. However, a possible explanation of this frameshifting phenotype according to the peptidyl-tRNA slippage model, would be that a fraction of the ${\text{tRNA}}_{\text{mnm}5\text{s}2\text{UUU}}^{\text{Lys}}$ is not available for translation. If so, it would decrease the concentration of active ternary complex containing Lys-${\text{tRNA}}_{\text{mnm}5\text{s}2\text{UUU}}^{\text{Lys}}$ and thereby inducing a ribosomal pause and thus frameshifting according to this model. One way to reduce the available concentration of ${\text{tRNA}}_{\text{mnm}5\text{s}2\text{UUU}}^{\text{Lys}}$ for translation would be that the geranylated tRNA is present in another compartment than the cytoplasm where translation occurs. We therefore tested the possibility that the geranylated tRNA was associated with the hydrophobic membrane. However, we did not find any evidence for an enrichment of geranylated tRNA in the membrane ruling out this possibility to explain the frameshifting phenotype at lysine codons. Another model to explain the frameshifting phenotype induced by the geranylated and fully charged ${\text{tRNA}}_{\text{mnm}5\text{s}2\text{UUU}}^{\text{Lys}}$ would be that the ternary complex containing geranylated lys-${\text{tRNA}}_{\text{mnm}5\text{s}2\text{UUU}}^{\text{Lys}}$ has a reduced decoding activity of the lysine codon AAA and thereby inducing a peptidyl-tRNA slippage. Indeed, geranylated 2-thiothymidine base pairs poorly to A but fairly well to G compared to the non geranylated derivative [40]. Accordingly, the translation rate of Glu codon GAA is more reduced by geranylation of ${\text{tRNA}}_{\text{mnm}5\text{s}2\text{UUC}}^{\text{Glu}}$ than its reading of Glu codon GAG [8]. Therefore, geranylation of ${\text{tRNA}}_{\text{mnm}5\text{s}2\text{UUU}}^{\text{Lys}}$ may also cause a slow decoding of AAA in the A-site, which would in turn induce a frameshift event according to the peptidyl slippage model. Alternatively, the ternary complex containing the geranylated ${\text{tRNA}}_{\text{mnm}5\text{s}2\text{UUU}}^{\text{Lys}}$ is inactive and this will reduce the concentration of a functional ternary complex containing non-geranylated ${\text{tRNA}}_{\text{mnm}5\text{s}2\text{UUU}}^{\text{Lys}}$ and thereby reducing the reading of the AAA codon. This will induce a pause and thereby a frameshifting event in the P-site. In summary, there are several ways to explain the frameshifting activity induced by fully charged geranylated ${\text{tRNA}}_{\text{mnm}5\text{s}2\text{UUU}}^{\text{Lys}}$ to be consistent with the peptidyl-tRNA slippage model. Still, a different mechanistic explanation not in line with the peptidyl-tRNA slippage model cannot be ruled out.

Chemically synthesized mnm^5^ges^2^U serves as a starting molecule to generate the selenium derivative mnm^5^Se^2^U by adding only sodium hydroselenide (NaSeH) in an ethanol solution [41]. Thus, a spontaneous chemical conversion occurs to generate mnm^5^se^2^U from mnm^5^ges^2^U. This would suggest that a linear conversion of mnm^5^s^2^U → mnm^5^ges^2^U → mnm^5^Se^2^U (Fig 1, alternative II) is operating to replace sulfur of mnm^5^s^2^U by selenium and that mnm^5^ges^2^U serves as an intermediate in the selenation reaction. If a linear reaction mechanism is operating (Fig 1, Alternative II) inhibition of the selenation reaction would result in an increased level of the potential intermediate geranylated tRNA. In a *selD* mutant selenation is blocked due to the lack of the selenium donor (SePO_4_), still the level of geranylation is about the same as in a *selD*^*+*^ strain ([4], data not shown). Moreover, a purified MnmH protein catalyzes the formation of the selenium derivative mnm^5^Se^2^U *in vitro*, without the presence of geranylpyrophosphate (GePP), which is the normal donor of a geranyl group [9]. These results would argue against any involvement of a geranylated intermediate in the selenation reaction. However, the selenium concentration in the growth medium might be very low, why a block in the synthesis of the selenium donor SePO_4_ by deleting the *selD* gene may not in a significant way further reduce the concentration of the selenium donor. This may explain why we did not observe any change in geranylation caused by lack of SelD even if a linear reaction mechanism is operating (Fig 1, Alternative II). Moreover, as shown in Fig 6 purified MnmH enzyme is enriched with geranylated tRNA, why such tRNA was likely present also in the purified enzyme preparations used by Wolfe *et al* [9]. Therefore, the geranylated tRNA bound to their purified MnmH enzymes may serve as an intermediate for selenation if a linear process according to Fig 1 (Alternative II) is operating explaining why GePP is not required in their selenation *in vitro* [9]. Wolfe *et al* [9] also added tRNA prepared from a *ΔmnmH* strain and such tRNA contains no geranylated or selenated derivative. Omitting such tRNA in the reaction mixture, the selenation is reduced by 52% as compared when it is present and increasing the amount of *ΔmnmH* tRNA also increases the extent of selenation. Thus, in some way *ΔmnmH* tRNA added to the reaction mixture stimulates selenation although no GePP was added [9]. This would suggest a more complex mechanism than a linear process as outlined in Fig 1 alternative II. However, since the MnmH protein has a strong affinity for geranylated tRNA (Fig 6), the purified MnmH enzyme used in selenation *in vitro* may also contain the substrate GePP, which would allow geranylation of the added *ΔmnmH* tRNA which then serves as an intermediate in selenation. The chemical mechanism of geranylation and selenation is likely to be different. Still, the rhodanese domain is important for both reactions, suggesting that although mechanistically different, both reactions may occur on this domain perhaps without the intermediate geranylated tRNA leaving the enzyme. These results are consistent with a geranylated tRNA as intermediate in selenation. The generation of (c)mnm^5^ges^2^U34 may result in a leaving group, which facilitates the exchange of the ges^2^-group by selenium. Clearly, more detailed studies are required to elucidate the details of the mechanism of selenation.

## Supporting Information

S1 Table Strains and plasmids. (DOCX)

S1 Fig HPLC. (EPS)

S2 Fig Growth. (EPS)

S3 Fig Northern. (EPS)
