# Supplementary material for: Transfer RNA Bound to MnmH Protein Is Enriched with Geranylated tRNA – A Possible Intermediate in Its Selenation?
Source: PLoS One. 2016 Apr 13;11(4):e0153488. doi: 10.1371/journal.pone.0153488 (PMC4830565; doi:10.1371/journal.pone.0153488)
Supplement: S1 Table — (DOCX) [file pone.0153488.s001.docx]

**Table S1**. Strains and plasmids

| Strain | Genotype | References |
| --- | --- | --- |
| ***Salmonella enterica* serovar Typhimurium** | |  |
| LT2 | *wt* | Laboratory collection |
| GT945 | *hisO1242, hisC3737, mnmH205*(G67R) | Laboratory collection |
| GT946 | *hisO1242, hisC3737, mnmH206* (G67R) | Laboratory collection |
| GT1484 | *hisO1242, hisC3737, mnmH204*(G67E) | Laboratory collection |
| GT5687 | *mnmH204*(G67E)*, zbb-2523::*Tn*10d*Tc | Laboratory collection |
| GT5688 | *zbb-2523::*Tn*10d*Tc | Laboratory collection |
| GT6315 | pKD46/LT2 | Laboratory collection |
| GT6680 | *mnmH183*<>Km | Laboratory collection |
| GT6682 | *mnmH183*<>FRT | Laboratory collection |
| GT6874 | *hisC3737,hisO1242, mnmH183<>*FRT | Laboratory collection |
| GT7321 | *hisO1242, hisD10122* (CCC-CAA), *zdd-2532*::Cm | Laboratory collection |
| GT7484 | *mnmH219*(G67R) | Laboratory collection |
| GT8187 | *hisD10122*, *hisO1242*, *mnmH193*(G67I),*zbb-2523*::Tn*10d*Tc, *zdd-2532*::Cat | This study |
| GT8188 | *hisD10122*, *hisO1242*, *mnmH194*(G67T),*zbb-2523*::Tn*10d*Tc, *zdd-2532*::Cm | This study |
| GT8189 | *hisD10122*, *hisO1242*, *mnmH195*(G67L),*zbb-2523*::Tn*10d*Tc, *zdd-2532*::Cm | This study |
| GT8190 | *hisD10122*, *hisO1242*, *mnmH196*(G67R),*zbb-2523*::Tn*10d*Tc, *zdd-2532*::Cm | This study |
| GT8191 | *hisD10122*, *hisO1242*, *mnmH197*(G67N),*zbb-2523*::Tn*10d*Tc, *zdd-2532*::Cm | This study |
| GT8192 | *hisD10122*, *hisO1242*, *mnmH198*(G67C),*zbb-2523*::Tn*10d*Tc, *zdd-2532*::Cm | This study |
| GT8193 | *hisD10122*, *hisO1242*, *mnmH199*(G67H),*zbb-2523*::Tn*10d*Tc, *zdd-2532*::Cm | This study |
| GT8194 | *hisD10122*, *hisO1242*, *mnmH200*(G67V),*zbb-2523*::Tn*10d*Tc, *zdd-2532*::Cm | This study |
| GT8195 | *hisD10122*, *hisO1242*, *mnmH181*(G67M),*zbb-2523*::Tn*10d*Tc, *zdd-2532*::Cm | This study |
| GT8216 | *mnmH220*(G67E)*, hisO1242, zdd-2532*::Cm*, zbb-2523*::Tn*10d*Tc, *hisD10122* | This study |
| GT8218 | *hisO1242, hisD10122* (CCC-CAA), *zdd-2532*::Cm, *mnmH185*(R129C) | This study |
| GT8219 | *hisO1242, hisD10122* (CCC-CAA), *zdd-2532*::Cm, *mnmH186(*G67E,R69Q) | This study |
| GT8220 | *hisO1242, hisD10122* (CCC-CAA), *zdd-2532*::Cm, *mnmH187(*R79W) | This study |
| GT8221 | *hisO1242, hisD10122* (CCC-CAA), *zdd-2532*::Cm, *mnmH188(*A63P) | This study |
| GT8223 | *hisO1242, hisD10122* (CCC-CAA), *zdd-2532*::Cm, *mnmH189(*L66P) | This study |
| GT8227 | *hisO1242, hisD10122* (CCC-CAA), *zdd-2532*::Cm, *mnmH191(*G67R) | This study |
| GT8228 | *hisO1242, hisD10122* (CCC-CAA), *zdd-2532*::Cm, *mnmH192(*G67V) | This study |
| GT8229 | *hisO1242, hisD10122* (CCC-CAA), *zdd-2532*::Cm, *mnmH190(*A82T) | This study |
| GT8243 | *hisO1242,hisC3737,* *mnmH182*(G67E,K155A) | This study |
| GT8248 | pUST317/ *mnmH183*<>Km | This study |
| GT8253 | *mnmC19*<>Km, *mnmH183*<>FRT | This study |
| GT8269 | *hisO1242, hisC3737, mnmH204(*G67E*), mnmC19*<>Km | This study |
| GT8270 | *mnmC19*<>Km*, mnmH182(*G67E, K155A*)*, | This study |
| GT8271 | *hisO1242, hisC3737, mnmH204(*G67E*), mnmE17*<>Km | This study |
| GT8272 | *mnmE17*<>Km, *mnmH183*<>FRT | This study |
| GT8273 | pUST315/ *mnmE17*<>Km, *mnmH183*<>FRT | This study |
| GT8277 | zbb2523*::*Tn*10d*Tc, *mnmH183*<>FRT | This study |
| GT8279 | pUST315/ *mnmH183*<>Km | This study |
| GT8286 | *mnmH222* (G67E, C96S),*hisO1242, hisD10122, zdd-2532*::Cm, *sfbA-2537*-MudSac | This study |
| GT8287 | *mnmH221* (G67E, C97S),*hisO1242, hisD10122, zdd2532*::Cm, *sfbA-2537*-MudSac | This study |
| GT8289 | *hisO1242, hisC3737, mnmH204,* | This study |
| ***Escherichia coli*** | |  |
| GRB1487 | (LMG194) F- ∆*lacX*74 *galE* *thi* *rpsL* ∆*pho*A (Pvu II) *∆ara714* *leu*::Tn*10d*Tc | From L.M. Guzman |
